# *Neptunomyces aureus* gen. et sp. nov. (Didymosphaeriaceae, Pleosporales) isolated from algae in Ria de Aveiro, Portugal

**DOI:** 10.3897/mycokeys.60.37931

**Published:** 2019-10-31

**Authors:** Micael F.M. Gonçalves, Tânia F.L. Vicente, Ana C. Esteves, Artur Alves

**Affiliations:** 1 Department of Biology, CESAM, University of Aveiro, 3810-193 Aveiro, Portugal Universidade de Aveiro Aveiro Portugal; 2 Universidade Católica Portuguesa, Institute of Health Sciences (ICS), Centre for Interdisciplinary Research in Health (CIIS), Viseu, Portugal Universidade Católica Portuguesa Viseu Portugal

**Keywords:** Didymosphaeriaceae, marine fungi, phylogeny, taxonomy

## Abstract

A collection of fungi was isolated from macroalgae of the genera *Gracilaria*, *Enteromorpha* and *Ulva* in the estuary Ria de Aveiro in Portugal. These isolates were characterized through a multilocus phylogeny based on ITS region of the ribosomal DNA, beta-tubulin (*tub2*) and translation elongation factor 1 alpha (*tef1-α*) sequences, in conjunction with morphological and physiological data. These analyses showed that the isolates represented an unknown fungus for which a new genus, *Neptunomyces***gen. nov.** and a new species, *Neptunomyces aureus***sp. nov.** are proposed. Phylogenetic analyses supported the affiliation of this new taxon to the family Didymosphaeriaceae.

## Introduction

The family Didymosphaeriaceae is an important family in the order Pleosporales introduced by Munk (1953) and typified by the genus *Didymosphaeria* Fuckel with *D. epidermidis* as the type species. Members of this family are characterized by having brown 1-septate ascospores and trabeculate pseudoparaphyses that anastomose mostly above the asci (Aptroot 1995, Hyde et al. 2013, Ariyawansa et al. 2014a, b). Species of Didymosphaeriaceae are saprobes, endophytes or pathogens of a wide variety of plant species worldwide (Ariyawansa et al. 2014a, Liu et al. 2015, Wanasinghe et al. 2016).

Accurate species’ identification in genera of the family Didymosphaeriaceae was discussed in detail by Ariyawansa et al. (2014a). Phylogenetic analyses based on regions such as the internal transcribed spacer (ITS) region of the ribosomal DNA, beta-tubulin (*tub2*) and translation elongation factor 1 alpha (*tef1-α*) proved to be useful in delimiting taxa (Tennakoon et al. 2016, Ariyawansa et al. 2014b). Several studies have been conducted to resolve the boundaries of this family. First, Ariyawansa et al. (2014a) showed that *Montagnulaceae* and Didymosphaeriaceae were synonyms and thus, Ariyawansa et al. (2014b) synonymized *Montagnulaceae* under Didymosphaeriaceae and rearranged the family into 16 genera: *Alloconiothyrium*, *Barria*, *Bimuria*, *Deniquelata*, *Didymocrea*, *Didymosphaeria*, *Julella*, *Kalmusia*, *Karstenula*, *Letendraea*, *Montagnula*, *Neokalmusia*, *Paraconiothyrium*, *Paraphaeosphaeria*, *Phaeodothis* and *Tremateia*. Subsequently, in the last years, additional genera were added, namely *Paracamarosporium* and *Pseudocamarosporium* (Wijayawardene et al. 2014), *Spegazzinia* (Tanaka et al. 2015), *Xenocamarosporium* (Crous et al. 2015), *Austropleospora* and *Pseudopithomyces* (Ariyawansa et al. 2015) and *Laburnicola* and *Paramassariosphaeria* (Wanasinghe et al. 2016). More recently, Jayasiri et al. (2019) introduced *Cylindroaseptospora* and Gonçalves et al. (2019) reassigned the genus *Verrucoconiothyrium* previously included in the family Didymosphaeriaceae to the family *Didymelaceae*. Thus, the family Didymosphaeriaceae currently comprises 25 genera.

During an extensive survey of the fungal diversity from macroalgae species in the salt marsh of Ria de Aveiro in Portugal, we gathered a collection of fungal isolates. Here we report the morphological, cultural and phylogenetic characterization of these fungal isolates and introduce a novel genus and species to accommodate them.

## Material and methods

### Collection and isolation

Macroalgae (*Gracilaria gracilis*, *Enteromorpha intestinalis*, and other macroalgae species identified at genus-level only) were collected from various sites in the estuary Ria de Aveiro in Portugal (Table 1). Samples were placed in sterile plastic containers and maintained at 4 °C until fungal isolation. Algae samples were washed with autoclaved filtered saline water, cut into small pieces and placed on Potato Dextrose Agar (PDA) enriched with 3 % (w/v) sea salts (Sigma-Aldrich). Streptomycin and tetracycline, at final concentrations of 100 mg/L, were added to PDA to inhibit the growth of bacteria. From each sample (algae) 20 pieces of tissue were plated on PDA medium. The plates were incubated at 25 °C for 5 days and examined daily to observe the growth of fungal hyphae. Distinct fungal colonies were then transferred to new PDA plates for further isolation and purification.

**Table 1.** Sampling sites.

Locality name	GPS coordinates	Sampling date	Algae species collected
Ria de Aveiro	40°37'45"N, 8°43'27"W	26/09/18	*Ulva* sp.
	40°39'33"N, 8°43'27"W		*Enteromorpha* sp.
	40°40'38"N, 8°42'20"W		*Gracilaria gracilis*, *Ulva* sp.
	40°43'00"N, 8°42'04"W		*Enteromorpha intestinalis*, *Ulva* sp.

### DNA isolation, amplification and analyses

Genomic DNA was extracted from fresh mycelium of cultures growing on PDA according to Möller et al. (1992). The primers ITS1 and ITS4 (White et al. 1990) were used for ampliﬁcation and sequencing of the ITS region of the ribosomal DNA was as described by Alves et al. (2004). Beta-tubulin (*tub2*) gene was ampliﬁed and sequenced using T1 and Bt2b primers (Glass and Donaldson 1995, O’Donnell and Cigelnik 1997) with the cycling conditions previously described by Lopes et al. (2017). Translation elongation factor 1 alpha (*tef1-α*) gene was amplified and sequenced using EF1-688F and EF1-2218R primers (Rehner 2001, Alves et al. 2008). The ampliﬁed PCR fragments were puriﬁed with the NZYGelpure kit (NZYTech, Portugal) before sequencing at GATC Biotech (Cologne, Germany). The nucleotide sequences were analyzed with FinchTV v.1.4.0 (Geospiza Inc. www.geospiza.com/ﬁnchtv). A BLASTn search against the nucleotide collection (nr/nt) database using the ITS, *tub2* and *tef1-α* sequences was carried out to determine the closest matching sequences, which were added to the sequence alignment. Sequences were aligned with ClustalX v. 2.1 (Thompson et al. 1997), using the following parameters: pairwise alignment parameters (gap opening = 10, gap extension = 0.1) and multiple alignment parameters (gap opening = 10, gap extension = 0.2, transition weight = 0.5, delay divergent sequences = 25 %). Alignments were checked and edited with BioEdit Alignment Editor v.7.2.5 (Hall 1999). Phylogenetic analyses were done with MEGA7 v.7.0 (Kumar et al. 2016). All gaps were included in the analyses. MEGA7 v.7.0 was also used to determine the best substitution model to be used to build the Maximum Likelihood (ML) tree. ML analysis was performed on a Neighbour-Joining (NJ) starting tree automatically generated by the software. Nearest-Neighbour-Interchange (NNI) was used as the heuristic method for tree inference with 1,000 bootstrap replicates. The sequences generated in this study were deposited in GenBank and taxonomic novelties in MycoBank. Alignment and tree were deposited in TreeBase (TB2:S24556).

### Morphology and growth studies

Observations of morphological characters were made with a SMZ1500 stereoscopic microscope (Nikon, Japan) and a Nikon Eclipse 80i microscope (Nikon, Japan) equipped with differential interference contrast. Fungal structures were mounted in 100% lactic acid. Photographs and measurements were taken with a Nikon DSRi1 camera (Nikon, Japan) and the NIS-Elements D program (Nikon, Japan). Colony characters and pigment production were registered after 2 weeks of growth on PDA, Malt Extract Agar (MEA) and Oatmeal Agar (OA) incubated at 25 °C. Colony colors (obverse and reverse) were assessed according to the color charts of Rayner (1970). Morphological descriptions were based on cultures sporulating on PDA and pine needles, after 1-month incubation at 25 °C.

Temperature growth studies were performed for the new species described. A 5-mm diameter plug was taken from the margin of an actively growing colony (14-day-old) and placed in the center of PDA, MEA and OA plates. Three replicate plates per isolate were incubated at 10, 15, 20, 25, 30 and 35 °C in the dark. Colony diameter was measured after 1 and 2 weeks.

To evaluate the growth requirements for sea salts, the new species was cultured in PDA with 3% (w/m) sea salts. Three replicate plates per isolate were incubated at 25 °C for 2 weeks in the dark. After incubation the diameter of the colonies was measured and compared.

## Results

### Phenotype

Regarding conidial morphology, the fungal isolates studied were characterized by being aseptate and subcylindrical with rounded apices golden yellow conidia. For all media tested, the minimum, maximum and optimal growth temperatures were 10, 30 and 25 °C, respectively. No differences were observed in terms of colony diameter when grown in PDA with and without the addition of 3% sea salts, indicating that this fungus does not require salt for growth.

### Phylogenetic analysis

BLASTn searches against the NCBI nucleotide database using the ITS, *tub2* and *tef1-α* sequences of the isolates retrieved various hits, of which those with the highest sequence similarity belonged to members of the family Didymosphaeriaceae. Based on a megablast search using the ITS sequence, the closest matches for MUM 19.38 = CMG 10A in GenBank were *Dothideomycetes* sp. (GenBank accession: HQ631008; Identities 549/564 (97%), no gaps) and *Letendraea* sp. (GenBank accession: LT796897; Identities 548/564 (97%), no gaps). The closest hits using the *tub2* sequence were *Letendraea* sp. (GenBank accession: LT796988; Identities 457/516 (89%), 5 gaps). Closest hits using *tef1-α* sequence also had highest similarity to *Letendraea* sp. (GenBank accession: LT797101; Identities 935/957 (98%), no gaps).

**Figure 1.**
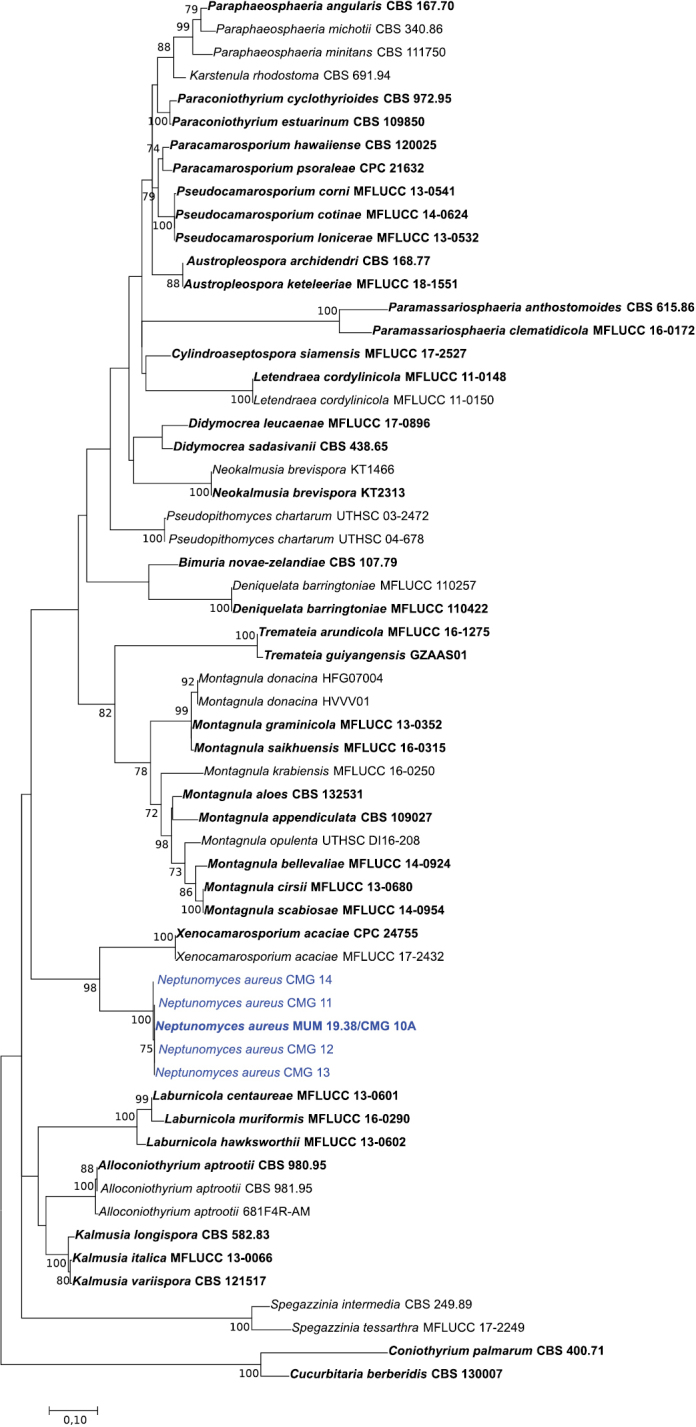
Phylogenetic relationships of Didymosphaeriaceae species based on ITS sequence data and inferred using the Maximum Likelihood method under the Kimura 2-parameter model. The tree is drawn to scale, with branch lengths measured in the number of substitutions per site and rooted to *Cucurbitaria berberidis* (CBS 130007) and *Coniothyrium palmarum* (CBS 400.71). Bootstrap values (> 70%) are shown at the nodes. Ex-type strains are in bold and the isolates from the current study are in blue.

**Figure 2.**
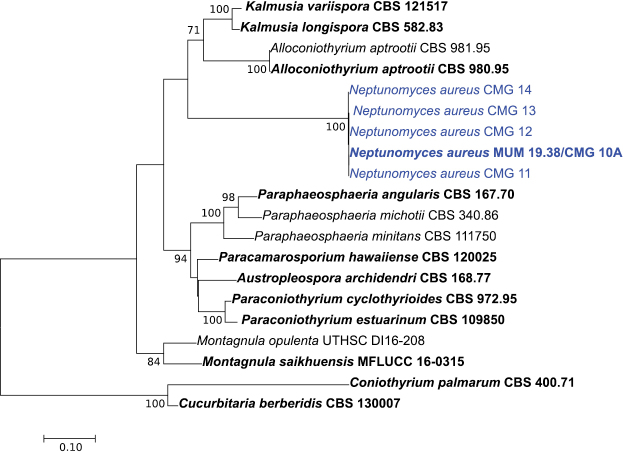
Phylogenetic relationships of Didymosphaeriaceae species based on ITS and *tub2* sequence data and inferred using the Maximum Likelihood method under the Kimura 2-parameter model. The tree is drawn to scale, with branch lengths measured in the number of substitutions per site and rooted to *Cucurbitaria berberidis* (CBS 130007) and *Coniothyrium palmarum* (CBS 400.71). Bootstrap values (> 70%) are shown at the nodes. Ex-type strains are in bold and the isolates from the current study are in blue.

**Figure 3.**
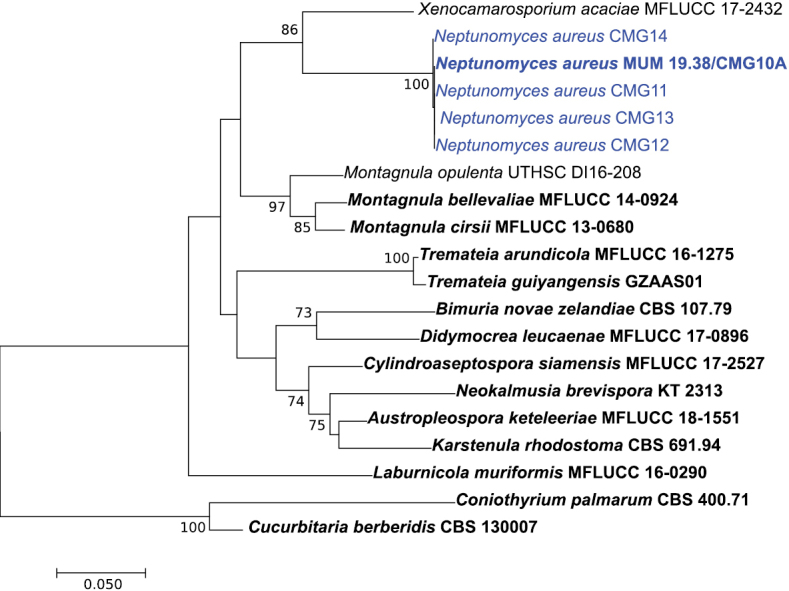
Phylogenetic relationships of Didymosphaeriaceae species based on ITS and *tef1-α* sequence data and inferred using the Maximum Likelihood method under the Kimura 2-parameter model. The tree is drawn to scale, with branch lengths measured in the number of substitutions per site and rooted to *Cucurbitaria berberidis* (CBS 130007) and *Coniothyrium palmarum* (CBS 400.71). Bootstrap values (> 70%) are shown at the nodes. Ex-type strains are in bold and the isolates from the current study are in blue.

To confirm the phylogenetic placement of the fungal isolates within the family Didymosphaeriaceae, sequences of ITS, ITS + *tub2* and ITS + *tef1-α* were aligned against those of several genera/species belonging to Didymosphaeriaceae (Suppl. material 1: Table S1). The alignment of the ITS, ITS + *tub2* and ITS + *tef1-α* contained 60, 20 and 20 sequences (including the outgroup), and there was a total of 1010, 1352 and 1836 positions in the final dataset, respectively. In all ML phylogenetic trees (Figs 1–3), all novel isolates clustered in a monophyletic clade that received high (100 %) bootstrap support within the family Didymosphaeriaceae with a close relationship with the genera *Alloconiothyrium* and *Kalmusia* (ITS + *tub2*, Fig. 2) and *Xenocamarosporium* (ITS + *tef1-α*, Fig. 3). Thus, this novel lineage is phylogenetically well delimited, and it is clearly distinct from the other genera of Didymosphaeriaceae described so far and therefore it is proposed here as a new genus and a new species.

### Taxonomy

#### Neptunomyces

Taxon classificationFungiPleosporalesDidymosphaeriaceae

M. Gonçalves, T. Vicente & A. Alves. Portugal gen. nov.

90966B60-2CC7-53F6-AA0B-D9C495E39648

831436

##### Description.

Asexual morph: mycelium consisting of septate, smooth hyphae, thick-walled, hyaline and rarely with nucleus. Conidia aseptate, golden yellow, smooth, subcylindrical with rounded apices. Chlamydospores not observed. Sexual morph unknown.

##### Etymology.

Referring to Neptune (Latin: *Neptūnus*) the god of the seas in Roman mythology.

##### Type species.

*Neptunomyces aureus* M. Gonçalves, T. Vicente & A. Alves. Portugal

#### Neptunomyces aureus

Taxon classificationFungiPleosporalesDidymosphaeriaceae

M. Gonçalves, T. Vicente & A. Alves. Portugal sp. nov.

68ED7DCA-10D4-5A69-AEC1-6A277A9900DA

831437

Fig. 4

##### Type.

Portugal, Ria de Aveiro (40°40'38"N, 8°42'21"W), isolated from *Gracilaria gracilis*, 26^th^ September 2018, M. Gonçalves, (holotype: a dried culture sporulating on pine needles AVE-F-1; ex-type living culture, MUM 19.38 = CMG 10A).

##### Etymology.

Referring to the golden yellow conidia.

##### Diagnosis.

Phylogenetic analysis based on the ITS, ITS and *tub2* and ITS and *tef1-α* dataset considered in the present study clustered the retrieved strains in a monophyletic lineage in the family Didymosphaeriaceae. Therefore, a new genus *Neptunomyces* gen. nov., and a new species *Neptunomyces aureus* sp. nov. are here proposed.

##### Description.

Mycelium smooth, white, 2–3 μm wide hyphae. Hyphae thick-walled, smooth, hyaline and rarely with nucleus. Conidiomata aggregated or solitary, globose to subglobose, dark brown, immersed or rarely superficial. Conidiomata wall pseudoparenchymatous. Conidiophores reduced to ampulliform to subcylindrical, hyaline, smooth conidiogenous cells (mean ± S.D. = 5.2 ± 0.3 × 2.0 ± 0.6 μm, n = 20). Conidia solitary, subcylindrical with rounded apices, aseptate, initially hyaline, smooth, becoming golden yellow (mean ± S.D. = 7.0 ± 0.6 × 2.7 ± 0.2 μm, n = 100). Sexual morph unknown.

**Figure 4.**
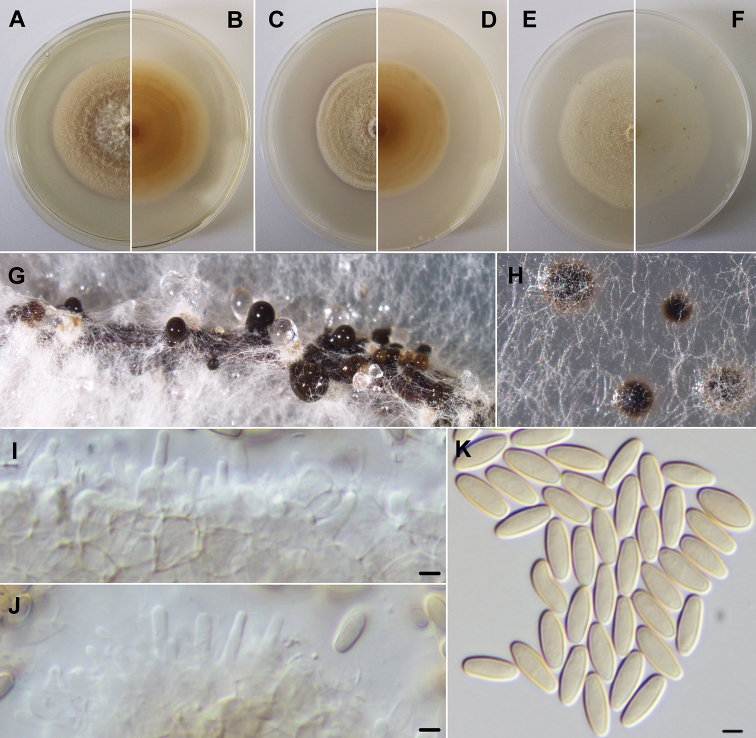
*Neptunomyces aureus* (MUM 19.38). **A, B** Colony after 2 weeks at 25 °C on PDA (obverse and reverse) **C, D** colony after 2 weeks at 25 °C on MEA (obverse and reverse) **E, F** colony after 2 weeks at 25 °C on OA (obverse and reverse) **G, H** conidiomata after 1 month at 25 °C on pine needles and PDA. **I, J** conidiogenous cells **K** conidia. Scale bars: 2.5 μm.

##### Culture characteristics.

On 2 weeks old PDA and OA plates, at 25 °C, colonies growing to 50 mm in diameter, regular and above and a little immersed into agar. PDA obverse white near the center getting flesh orange towards the borders; reverse buff orange in the center and lighter in periphery. OA obverse skimmed milk white; reverse snow white. On 2 weeks old MEA plates, at 25 °C, colonies growing to 44 mm in diameter, regular and above and a little immersed into agar. Obverse orange-colored white; reverse reddish orange in the center and ochre yellow in periphery. At 35 °C, there was no growth in any media tested.

##### Distribution.

Estuary Ria de Aveiro, Portugal

##### Additional specimens examined.

Portugal, Ria de Aveiro (Table 1), isolated from *Ulva* sp., *Enteromorpha intestinalis* and *Enteromorpha* sp. (Supp. material 1: Table S1). M. Gonçalves, living cultures CMG 11, CMG 12, CMG 13 and CMG 14.

##### Notes.

*Neptunomyces aureus* clustered in a distinct lineage in the family Didymosphaeriaceae with high p-distances (= 0.07) of nucleotide sites among the two-loci sequences (ITS and *tef1-α*) with closest genus *Xenocamarosporium*. Although the morphology of conidiomata, conidiomata wall and conidiogenous cells can be very similar in the genera of this family, conidial morphology distinguishes *Neptunomyces* from *Xenocamarosporium* (Table 2).

**Table 2.** Comparison of *Neptunomyces aureus* and *Xenocamarosporium acaciae*.

Species		*Neptunomyces aureus*	*Xenocamarosporium acaciae*
**Strain**		MUM 19.38	CBS 139895
**Nucleotide differences**	ITS	65	
	*tef1-α*	58	
**(p-distance)**	ITS + *tef1-α*	0.07	
**Conidia**	Size (μm)	7.0 ± 0.6 × 2.7 ± 0.2	(11–)12–14(–15) × (3.5–)4(–5)
	Morphology	Subcylindrical	Ellipsoidal to subcylindrical
	Apex and base	Rounded	Obtuse and rounded to truncate base
	Color	Hyaline becoming golden yellow	Hyaline becoming golden brown
	Septation	Aseptate	(1–)3-septate
**Conidiogenous cells**	Size (μm)	5.2 ± 0.3 × 2.0 ± 0.6	7–12 × 5–7
	Morphology	Ampulliform	Ampulliform
	Color	Hyaline	Hyaline
**References**		Present study	Crous et al. 2015

## Discussion

This study adds to the family Didymosphaeriaceae a new genus/species, namely *Neptunomyces aureus* isolated from macroalgae in the estuary of Ria de Aveiro in Portugal. The family Didymosphaeriaceae contains now 26 genera described.

The majority of the genera in the Didymosphaeriaceae remain under studied, which makes the family still poorly understood and not well resolved (Wanasinghe et al. 2016). In fact, there was no β-tubulin and *tef1-α* sequence data available for many species and therefore the phylogenetic analyses presented did not encompass all known species of the family. For example, phylogenetic analyses based on ITS + *tub2* revealed that *N. aureus* is closely related to the genera *Alloconiothyrium* and *Kalmusia*, while on ITS + *tef1-α* it is related to the genus *Xenocamarosporium*, since there is no *tef1-α*/*tub2* for *Alloconiothyrium*, *Kalmusia* and *Xenocamarosporium*, respectively. However, this family contains several well supported clades, most of which correspond to monotypic genera (*e.g. Alloconiothyrium*, *Bimuria*, *Karstenula*, *Xenocamarosporium*), or genera with only two species (*e.g. Cylindroaseptospora*, *Deniquelata*, *Didymocrea*).

Comparison of the ITS and *tef1-α* sequences from *N. aureus* and the closest genus/species *X. acaciae* revealed 65 and 58 base pair differences, respectively, with high p-distances (= 0.07) supporting the establishment of *Neptunomyces* as a distinct genus. Although the morphology of conidiomata, conidiomata wall and conidiogenous cells are similar, the conidiogenous cells of *N. aureus* are smaller than those of *X. acaciae*. Also, both can be easily discriminated by their conidia morphology, color and size. The conidia of *N. aureus* are aseptate, subcylindrical with rounded apices and initially hyaline and soon become golden yellow, while conidia of *X. acaciae* are mostly tri-septate, ellipsoidal to subcylindrical, sometimes with truncate base and golden brown. Moreover, conidia of *N. aureus* are considerably smaller than those of *X. acaciae*.

*Neptunomyces aureus* was isolated from healthy tissues of the macroalgae analyzed, where it may occur as endophyte or epiphyte. Further investigations are essential for clarifying its biology, ecology, physiological characteristics and host-specificity. Moreover, we did not obtain any sexual morph for this new species and there is no molecular support to link possible sexual taxa.

So far, species of Didymosphaeriaceae seem to be cosmopolitan in distribution: they have been recorded from both temperate and tropical regions. Also, Didymosphaeriaceae have been found on various hosts and substrates, including plants, humans and soil, being regarded as saprobes, endophytes or pathogens of a wide variety of plant substrates worldwide (Ariyawansa et al. 2014a, Liu et al. 2015, Wanasinghe et al. 2016). However, most Didymosphaeriaceous genera occur on plants of more than 20 host families, the majority of them being monocotyledons and herbaceous plants, such as *Anacardiaceae*, *Asparagaceae*, *Asteraceae*, *Caprifoliaceae*, *Euphorbiaceae*, *Fagaceae*, *Lecythidaceae* and *Poaceae*. Reports of Didymosphaeriaceous species in marine/estuarine environments are almost non-existent. So far, this new genus/species has been found only in association with macroalgae species. Garzoli et al. (2018) reported, for the first time, some species within this family in *Padina pavonica*, a brown alga collected in the Mediterranean Sea: *Paraconiothyrium variabile*, *Paraphaeosphaeria neglecta* and another eight unidentified Didymosphaeriaceous species. Also, *Paraconiothyrium estuarinum* was isolated from sediments of an estuarine environment (Verkley et al. 2004) and *Paraphaeosphaeria michotii* from *Phragmites australis*, also typically found in estuaries (Eriksson 1967).

Physiological tests allowed us to characterize the retrieved isolates as a slight halophile as they grow equally well in the presence and absence of 3% sea salts. Information regarding NaCl tolerance is still poorly described in *Didymosphaeriaceous* species, but future studies related to tolerance to salinity in these organisms (especially in this new species) may provide physiological unique characteristics which may have some biotechnological potential.

## Supplementary Material

XML Treatment for Neptunomyces

XML Treatment for Neptunomyces aureus

## Supplementary materials

Supplementary material 1 Table S1. List of isolates used in this study Data type: species data https://binary.pensoft.net/file/353773This dataset is made available under the Open Database License (http://opendatacommons.org/licenses/odbl/1.0/). The Open Database License (ODbL) is a license agreement intended to allow users to freely share, modify, and use this Dataset while maintaining this same freedom for others, provided that the original source and author(s) are credited.Micael F.M. Gonçalves, Tânia F.L. Vicente, Ana C. Esteves, Artur Alves
